# Food Group Consumption and Nutrient Intake by Breastfeeding Women: Comparison to Current Dietary Guidelines and Nutrient Recommendations

**DOI:** 10.3390/nu17030375

**Published:** 2025-01-21

**Authors:** Ying Jin, Jane Coad, Louise Brough

**Affiliations:** 1 School of Health Sciences, College of Health, Massey University, Palmerston North 4442, New Zealand; y.jin@massey.ac.nz; 2 School of Food Technology and Natural Sciences, College of Sciences, Massey University, Palmerston North 4442, New Zealand; j.coad@massey.ac.nz

**Keywords:** nutrient intake, food groups, breastfeeding, diet alignment to guidelines, nutrient toxicity, food and nutrient adequacy

## Abstract

Background/Objectives: Optimal nutrition is essential for the health of breastfeeding women and their infants. This study aimed to assess food and nutrient intake and alignment with nutrition guidelines for breastfeeding women living in New Zealand. Methods: Seventy-six breastfeeding women were enrolled in the longitudinal Mother and Infant Nutrition Investigation study and completed a weighed four-day diet diary including supplement use at three months postpartum. The number of servings consumed for each food group were calculated based on the 2020 Eating and Activity Guidelines for New Zealand Adults. Nutrient intakes were compared to the nutrient reference values for Australia and New Zealand. Results: Overall, the percentages of women who met the recommended number of servings for fruits, vegetables, grain foods, meats and milk/milk products were 25%, 0%, 5%, 34%, and 13%, respectively. None of women met the current recommendations for all food groups. Many participants had intakes below the estimated average requirement or adequate intake and were at risk of nutrient inadequacy for vitamin E (55%), vitamin D (53%), manganese (61%), and selenium (55%). Conclusions: Breastfeeding women had a low alignment with the current dietary guidelines and were at risk of an inadequate intake of vitamin E, D, manganese, and selenium. Research to investigate the barriers and enablers of healthy food choices is needed.

## 1. Introduction

Maternal nutrition during the first year after childbirth (the postpartum period) is important to meet the demands of breastmilk quality and quantity [1,2] as well as mothers’ own physiological needs. After childbirth, women appear to prioritise the health of their newborn infants rather than their own, as dietary adequacy has been shown to decline from pregnancy to postpartum in studies of women at 40 days after childbirth [3] and around six months postpartum [3,4]. The further decline during the late postpartum period was also reported in first-time Australian mothers [5], low-income non-Hispanic black mothers [6], and UK and Spanish postpartum women [7,8]. A longitudinal study of postpartum women from the United States reported maternal diet quality remained low over the period during 3, 6, 9, 12, and up to 18 months postpartum, indicating that early intervention would be useful in improving maternal diet [1]. However, only one 24 h diet recall was used at each time point, which may not reflect the habitual intake. Studies in Italy, Australia, and Spain mostly used food frequency questionnaires with 12 to 75 food items [2,5,7,8], which have limited the respondents’ ability to report on the adequacy of their nutrient intake.

Maternal diet quality has been linked with specific adverse outcomes. A 2014 report from the Norwegian Mother and Child Cohort study of Nordic women showed that higher adherence to Nordic food guidelines or Nordic Nutrition Recommendations during pregnancy was associated with lower postpartum weight retention [9]. The latest systemic review article has highlighted the scarcity of evidence for examining a postpartum diet concerning maternal mental wellbeing, with six eligible studies included, finding that following a healthy postpartum diet lessens postpartum depression symptoms [10]. A cross-sectional survey of Chinese lactating women (n = 939) reported that limited dietary diversity was associated with an increased risk of postpartum depression [11].

Inadequate nutrient intakes during lactation have been linked to low breast milk production and the suboptimal nutritional composition of human milk [1,12], which may impact breastfed infants’ growth and development. Nutrient inadequacies in lactating mothers have been reported in both high-income and low–middle-income countries. For example, only half of the studied mothers met the United States Dietary Reference Intakes for vitamin A, D, choline, and docosahexaenoic acid [13]. Chinese lactating women were reported to have inadequate intakes of vitamin C, folate and dietary fiber [14], while only 57% of rural Indonesian lactating women met their micronutrient requirements [15]. To support women in achieving a nutritionally adequate diet, dietary guidelines have been developed to recommend minimum serving sizes, frequency, and healthy choices for the five core food groups in most countries [16,17,18]. In 2006, the New Zealand Ministry of Health published the evidence-based “Food and Nutrition Guidelines for Healthy Pregnant and Breastfeeding Women” to promote healthy dietary choices for an optimal outcome for mothers and their offspring [19]. The guidelines were updated in 2020 based on the Australian Dietary Guidelines [17].

Adherence to these guidelines has been explored among pregnant women. A 2020 systematic review of 18 observational studies across 10 countries reported overall low adherence to food guidelines during pregnancy, in particular, the intakes of vegetables and grain [20]. However, there were limited data on breastfeeding women. One study reported adherence to the healthy eating pyramid (a visual presentation of the Mediterranean diet) via a self-reported questionnaire and found that pregnant women had the highest adherence compared to breastfeeding and non-pregnant, non-lactating women [21]. In New Zealand, dietary adherence was only studied on pregnant women collected from the Growing Up in New Zealand study (GUiNZ) (a nationally representative sample of pregnant women, n = 5664, data collected during 2009–2010) [22].

During lactation, the requirements for many nutrients differ compared to non-pregnant, non-lactating women, due to the additional needs of breastfed infants via breast milk. The latest systematic review of 28 observational studies from developed countries reported that the nutrient intake of breastfeeding women (5–7 weeks postpartum) mostly adhered to the recommended US Dietary Reference Values (DRVs), with some nutritional inadequacies, including potassium and vitamin D (although the primary source of vitamin D is from skin exposure to ultraviolet B rays from the sun) [16]. The nutrient reference values (NRVs) for Australia and New Zealand (2006) provide recommendations for nutrient intake throughout the lifecycle [23].

To date, a recent cross-sectional analysis of the nutrition profile of pregnant women in a longitudinal Australian birth cohort also found inadequate intakes of calcium, iron, iodine, and folate based on a semi-quantitative food frequency questionnaire (FFQ) [24]. However, only two studies reported micronutrient intakes among lactating women in New Zealand. One study of 73 breastfeeding women (3 months postpartum) was carried out in the 1990s and found a substantial level of dietary inadequacy, including zinc, calcium, folate, and vitamin A [25]. Another study of 78 breastfeeding women (6–8 weeks postpartum) published in 2018 reported inadequate daily intakes of folate, selenium, iodine, and molybdenum [26].

No recent data are available on dietary and nutrient intakes among breastfeeding women in New Zealand. This study aimed to determine whether the diets of these women align with national dietary recommendations and to assess their nutrient adequacy.

## 2. Materials and Methods

The Mother and Infant Nutrition Investigation (MINI) study was an observational longitudinal cohort study spanning the first year postpartum. It was approved by the Health and Disability Ethics Committee (15/NTA/172) in December 2015 and registered with the Australia and New Zealand Clinical Trials Registry (ACTRN12615001028594) in October 2015.

### 2.1. Study Participants

A cohort of breastfeeding women residing in the Palmerston North area in New Zealand, was recruited from June 2016 to December 2017 into the longitudinal observational Mother and Infant Nutrition Investigation (MINI) study spanning the first postpartum year [27]. Women aged 16 years and older, who had given birth to a healthy term singleton infant aged less than three months and who were breastfeeding, were invited to join the study. Written informed consent was obtained from all participants.

### 2.2. Maternal Demographic Data

Maternal demographic data (including age, ethnicity, educational attainment, annual household income), parity, childbirth delivery method, and infant feeding modes were collected via a self-administered online questionnaire. Women who breastfed their infants fully since the child was born are referred to as exclusively breastfeeding in this study.

### 2.3. Dietary Assessment Method

Participants completed a weighed four-day diet diary (4DDD) [28] at around three months postpartum. The four days were consecutive and included one weekend day. Detailed food items, brands, amounts consumed, and the content of the nutritional information panel, if applicable, were recorded. All food and beverage items consumed were weighed with an electronic kitchen scale or measured using household measurement cups and spoons, which were provided. All women received oral and written instructions on completing the diary, including a one-day written example. Women were asked to record any dietary supplements consumed each time, including the brand and dose. Dietary data were analyzed using Foodworks 9 Professional (Xyris Software, Australia) using the dataset from New Zealand Foodfiles 2016. Where food items were not included in Foodfiles 2016, new food items were created based on the information directly provided by participants, or from appropriate international databases from Australia and the United States. A registered nutritionist (YJ) checked all dietary data to ensure their accuracy and completeness.

### 2.4. Dietary Adherence and Nutrient Intake Analysis

Dietary adherence was assessed by comparing the number of servings (NS) from daily food consumption in each food group to the revised 2020 Eating and Activity Guidelines for New Zealand Adults (EAGNZ) [19] (Table 1). The serving size was manually calculated for each food item reported in each individual’s four-day dietary diary using the suggested serving sizes from the EAGNZ. Adherence was defined as consuming or exceeding the recommended daily NS for each food group.

Nutrient intakes from food and supplements were compared to the latest nutrient reference values for Australia and New Zealand (2006—the most up-to-date version) [23] to assess dietary adequacy. For a population to have a low prevalence of inadequate dietary intake, the mean/median intake should be above the recommended daily intake (RDI), while the percentage below the estimated average requirement (EAR) approximates the proportion that is at risk of nutrient inadequacy according to the EAR cut-point method [29,30]. In the current study, the percentage below the EAR or adequate intake (AI) is used as a proxy to measure the proportion of the group with inadequate intake. The acceptable macronutrient distribution range (AMDR) was used to assess the macronutrient intake contributing to energy intake. Nutrient intakes for food and supplements were compared to the upper level of intake (UL) to ascertain the risk of toxicity. Dietary intakes of selenium and iodine have already been published elsewhere [31,32].

**Table 1:** Serving size recommendations for breastfeeding women and exclusion from the analysis.

Food Group	Foods in Group	Recommended Number of Servings Per Day ^1^	Specific Foods Excluded from the Analysis
Vegetables and fruits	Fresh, frozen, and canned vegetables and fruits	At least **7.5** servings of vegetables and **2** servings of fruits	Juice, dried fruit, and deep-fried potato chips
Grain foods	Bread, cereals, brown rice, pasta, noodles, rice, and couscous	At least **9** servings (choose wholegrain bread and cereals)	Cakes and biscuits
Milk and milk products	All dairy milk and milk products	At least **2.5** servings (choose low- or reduced-fat options)	Ice cream, alternative milks (rice, almond and soy)
Lean meat, meat alternatives, and eggs	Meat, poultry, eggs, fish, seafood, nuts, seeds, lentils, split peas, chickpeas, cooked dried kidney beans, and baked beans	At least **2.5** servings	Processed meats: sausages, ham, salami, luncheon, battered or deep-fried fish, and chicken nuggets

^1^ Ministry of Health (2020) Eating and Activity Guidelines for New Zealand Adults. Wellington, New Zealand [33].

### 2.5. Statistical Analysis

All data were analyzed using IBM SPSS (Statistics Package for the Social Sciences, IBM, Armonk, NY, USA) version 20, and the significance level was set at *p* < 0.05. Data were tested for normality using the Shapiro–Wilk test. Non-parametric data were expressed as median (25th, 75th percentile) and parametric data were expressed as mean (±standard deviation; SD). Differences in median intakes were investigated using the Mann–Whitney U test. Spearman’s correlation was used to examine the association between the daily NS consumed for each food group and nutrient intake. When analyzing the association between the daily NS consumed for the milk and milk products group and calcium intake, plant-based milks were excluded.

## 3. Results

Overall, 76 breastfeeding women took part in the study. Participants were predominantly Caucasian (77%), with 82% achieving tertiary education or higher. Of the participants, 45% were breastfeeding their first infant, and 65% were exclusively breastfeeding at three months postpartum (Table 2).

### 3.1. Food Groups’ Consumption and Alignment with Recommendations

None of the participants achieved all the recommended NS, and 37% did not achieve any of the recommendations. Overall, 63% of women met at least the recommended NS for one food group. Of the five food groups, the greatest alignment was with the recommendations for the lean meat, legumes, and eggs group, with 34% of the total sample meeting the minimum recommended servings for breastfeeding women (Table 3). Women with tertiary qualifications consumed a higher NS daily for grain foods (4.0 vs. 3.0, *p* = 0.001) and vegetables (3.0 vs. 2.2, *p* = 0.010) compared to those without tertiary education. None of the other maternal characteristics, including household income, parity, delivery methods, and infant feeding modes, showed any differences in NS intake for the five food groups.

The daily NS consumed in the vegetable group was weakly positively correlated with the daily NS consumed in the lean meat, legumes, and eggs group (r = 0.350, *p* = 0.002), but it was weakly negatively correlated with the daily NS consumed in the milk and milk products group (r = −0.246, *p* = 0.032).

Many women consumed foods that were not recommended for lactation in the EAGNZ. A third of women (32%) consumed alcohol, 28% had soft, flavored, or energy drinks, and 28–29% had fruit juice or dried fruit. In total, 68% of women ate processed meat, including luncheon meat, salami, ham, bacon, or sausages.

### 3.2. Nutrient Intake from Food and Supplements

Median energy intake was 9706 (8148, 10,968) kJ and median intakes of macronutrients as a percentage of energy were 16.6% protein, 37.9% total fat, 15.5% saturated/trans-fat, and 40.0% carbohydrate (Table 4). Most participant intakes were above the acceptable macronutrient distribution range (AMDR) for total fat (68%) and saturated/trans-fat (88%) and below the AMDR for carbohydrate (80%), with 22% below the protein recommendation. Median protein intake was 92.5 (81.7, 106.5) g, higher than the RDI of 67 g. Median fiber intake was 29 (21, 34) g, 55% below the adequate intake (AI) of 30 g.

Many participants had intakes below the EAR or adequate intake. They were at risk of nutrient inadequacy for micronutrients (Appendix A) for vitamin E (55%), vitamin D (53%), folate (42%), vitamin A (41%), thiamin (26%), vitamin C (24%), manganese (61%), selenium (55%), calcium (36%), potassium (34%), copper (33%), zinc (28%), and magnesium (11%).

Some participants had intakes from food and supplements above the upper level of intake (UL), including iron (12%, 45 mg/day) and folic acid (3%, 1000 mcg/day), and 5% of women had excessive intakes of vitamin C above the prudent UL of 1000 mg/day. No maternal characteristics were associated with nutrient intake.

### 3.3. Nutrient Intakes and Consumption of Food Groups

There was no significant association found between macronutrient intake and the consumption of food groups. Dietary fiber intake was weakly positively correlated with the daily NS consumed for vegetables (r = 0.295, *p* = 0.015), but not with other food groups. Calcium intake was weakly positively correlated with the daily NS consumed for milk and milk products (excluding plant-based milks) (r = 0.260, *p* = 0.034). Both selenium and iron intakes were weakly positively correlated with the daily NS consumed for the meat, eggs, and legume group, respectively (r = 0.333, *p* = 0.006; r = 0.307, *p* = 0.011).

## 4. Discussion

This research shows a low alignment with the Eating and Activity Guidelines for New Zealand Adults (EAGNZ) among this cohort of breastfeeding women. None of the women met the recommendations for all five food groups, which was lower than pregnant women reported in the nationally representative GUiNZ study, where 3% met all recommendations [22]. This is consistent with the findings from a cohort of Spanish breastfeeding women (n = 437) at an average of 7.8 months postpartum, who had low adherence to the healthy food pyramid compared to pregnant and non-pregnant non-lactating women [21]. A 2020 systematic review of 17 observational studies reported decreasing dietary adherence to recommendations from pregnancy to postpartum (40 days, 4 months, or 6 months postpartum) [34]. These may suggest that specific interventions are needed to improve dietary adherence in this population group.

No women in the current study met the recommendations for vegetables (four servings), which is of concern. The existing literature indicates health benefits and a significant impact on reducing mortality risk from consuming fruit and vegetables [35]. However, consuming adequate vegetables showed a favorable positive effect on psychological wellbeing compared to fruit consumption [36]. In GUiNZ, 27% of pregnant women consumed four or more servings of vegetables, which was higher than the current study. Even more concerningly, the recommended servings of vegetables are much higher now, up to 7.5 servings [19], which is not widely promoted, unlike the “5+ A day” initiative. A much higher number of women met the recommended servings of fruit in the GUiNZ (43%) than in the current study (25%). Achieving the recommended fruit intake is more common in both studies than meeting the recommendations for vegetables. Ethnicity was the strongest association among the examined demographic variables in GUiNZ; the authors suggested ethnic-specific interventions [22]. The diverse population in New Zealand must be considered when promoting fruit and vegetable consumption for all pregnant and lactating women.

Many factors contribute to low alignment with food-based dietary guidelines, including a lack of awareness of the updated guidelines, lack of access to healthy foods, and limited knowledge on achieving healthy meals by choosing alternatives based on the available budget [37]. The need to support their children, lack of family support, and financial limitations were specific barriers for postpartum women to meet healthy eating guidelines, based on a 2021 systematic review of 28 qualitative and quantitative studies [38]. In addition, fatigue, feeling stressed, and lack of time were the most commonly reported barriers in a cohort of UK postpartum women [8]. When developing interventions for postpartum women, effective communication of healthy dietary recommendations is needed. Multiple factors should be considered to improve women’s capability and to provide opportunities and motivation to support women in achieving healthy eating habits.

A substantial proportion of the present study population had macronutrient intakes outside of the recommendations, with a higher percentage of energy from total and saturated/trans-fat and a lower percentage of energy from carbohydrate and lower total fiber intakes, mirroring findings amongst lactating women in other developed countries based on a systemic review of 28 studies [39], in Spain [40], and in China [14], and the general adult population in New Zealand [41]. The protein intake being higher than the RDI is consistent with the rest of the NZ population and with breastfeeding women in other developed countries [39]. High saturated and trans-fat intakes have been linked to an increased cardiovascular risk [42].

The health benefits of consuming a high-dietary-fiber diet have been well-documented. These include improving insulin sensitivity, lipid profiles, gut microbial viability and diversity, and reducing abdominal adiposity [43]. Although the dietary fiber intake in this study cohort was higher than the median value of 23.4 g/d in developed countries [39], more than half of the cohort did not meet the AI. This is consistent with the low alignment with healthy eating recommendations, especially in the number of servings of vegetables and fruit consumed. Supporting lactating women in improving their vegetable and fruit consumption would ultimately increase their dietary fiber intake, benefiting women’s long-term health.

The current study suggests that participants were at risk of nutrient inadequacy during lactation for nine micronutrients (thiamin, folate, vitamins A, C, and E, selenium, magnesium, calcium, and zinc). Compared to a recently published multicenter European cohort (ATLAS) study, lactating women’s nutrient intakes of vitamins A, C, and D, folate, and iodine were below the dietary reference values (DRVs), based on a three-day dietary diary up to four months postpartum, regardless of their dietary patterns [44]. Low intakes of folate, vitamin A, calcium, and zinc were also reported in an early 1990s study of exclusively breastfeeding New Zealand women based on repeated 24 h recalls [25]. Many of these micronutrients are similar to those lacking in the diet of the general population, but in this study, a far more significant proportion were at risk of inadequate intake than previously seen in reproductive-aged women in New Zealand National Nutrition Surveys [41,45]. If this magnitude of dietary inadequacy exists, this would be very concerning. Biomedical studies are needed to confirm whether nutrient deficiency is a problem.

Even when supplements were included, 42% of women in our study had inadequate intake of folate, which was reported in the ATLAS study (the mean folate intake ranged between 51 and 89% of the DRV) [44]. The New Zealand situation may have improved since the mandatory fortification of bread with folic acid was implemented in mid-2023 in New Zealand [46], allowing equitable health outcomes. However, two women in our study had intakes of folic acid above the UL of 1000 µg/day, which is concerning. Implementing mandatory fortification of bread means women who take regular supplements containing folic acid may be at risk of excessive intake. Further monitoring of folic acid intake and status assessed via biomarkers are needed to ensure optimal health outcomes for mothers and their children. Some current study participants had excessive intakes of iron and vitamin C due to supplementation, which could have adverse consequences for maternal and infant health. Breastfeeding women must receive the correct advice regarding the use of supplements, and this is of particular concern when women use multiple micronutrient supplements.

## 5. Strengths and Limitations

To our knowledge, this is the first study to examine the alignment with the EAGNZ among breastfeeding women in New Zealand. The strength of this study was to collect robust dietary data via a weighed four-day dietary diary (4DDD), which is considered the gold standard for dietary assessment [47]. Participants were provided with clear instructions, both oral and written, which enabled detailed dietary information to be collected. Women were also asked to include any dietary supplements consumed in their 4DDD to reflect their dietary intake correctly. Goldberg cut-offs are commonly used in dietary research to identify misreporting [48] using a BMR based on Schofield equations [49]. However, there is currently no consensus on BMR variation in lactation, so we were unable to identify misreporting in the present study.

The small sample size of this study limits the applicability of these results to the wider population. This also limited the ability to analyze differences in food group choices from various ethnicities. For example, it is a well-known fact that most non-Caucasian adults are more likely to present lactose intolerance, and this can be the reason why Māori and Asian individuals do not consume those kinds of products. However, women in this study were better educated than the New Zealand population; if poor dietary alignment was found in this group, it is likely that women who are less educated and under-represented will be of concern. It is worth noting that our data were collected just before the publication of the updated 2020 Eating and Activity Guidelines New Zealand; participants would not know this information. However, knowing does not necessarily directly result in behavioral changes; other psychosocial factors acting as barriers may be considered towards adopting healthy eating habits [38]. Despite its limitations, this research provides a useful starting point from which more robust studies thoroughly investigating nutrient status via biological samples can be developed. Such studies will help to understand if dietary intakes are inadequate for many breastfeeding women or if nutrient recommendations require revision.

## 6. Conclusions

Breastfeeding women in this study had a low alignment with the EAGNZ, a high fat, low carbohydrate intake, and micronutrient inadequacy. The excessive use of supplements by some women is concerning. The current study contributed to the global understanding of food group and nutrient intakes among breastfeeding women from a high-income country. Studies of biomarkers are required to determine the incidence of micronutrient deficiency among breastfeeding women in New Zealand. Future research is needed to identify the barriers to healthy eating and to develop effective initiatives to encourage and support breastfeeding women to achieve healthy eating behaviors and to ensure adequate nutrient intake to optimize health outcomes for mothers and their infants.

## Figures and Tables

**Table 2:** Description of maternal characteristics (n = 76).

Maternal Characteristics	n (%)
Age, years (Mean ± SD)	32 ± 4
Education—Tertiary	62 (82)
Ethnicity—Māori	7 (9)
Ethnicity—Caucasian	59 (77)
Ethnicity—Asian ^2^	8 (11)
Annual household income (Above median) ^1^	49 (64)
Primiparity	34 (45)
Caesarean delivery	14 (18)
Exclusively breastfeeding	65 (86)

^1^ Median annual household income based on data from Statistics New Zealand was 75,995 New Zealand dollars for the year ending June 2016 [25]. ^2^ The remainder of the ethnicity data comprised Latin Americans.

**Table 3:** Median daily servings consumed from food groups and alignment with the serving recommendations.

Food Groups	Median Daily Servings (25th, 75th)	Achieving Recommendations n (%)
Vegetables	2.7 (1.7, 3.9)	0 (0)
Fruit	1.0 (1.0, 1.8)	19 (25)
Grain foods	4.0 (3.0, 5.0)	4 (5)
Milk and milk products ^1^	1.2 (0.8, 1.7)	10 (13)
Lean meat, legumes, and eggs	2.0 (1.5, 2.7)	26 (34)
At least one food group		48 (63)
None of food groups		28 (37)

^1^ This analysis includes the consumption of plant-based milks.

**Table 4:** Daily energy and macronutrient intake from food and supplements compared to the Australia and New Zealand nutrient reference values (n = 76).

	MINI Study			Reference Recommendations		
	Median Intake (25th, 75th)	n (%) <AMDR/EAR/AI	n (%) >AMDR	Australia and New Zealand ^1^	EFSA ^2^	NIH ^3^
Total energy (kJ/day)	9706 (8148, 10,968)			NA	AR (+) 2100 ^4^	NA
% energy protein	16.6 (15.1, 18.9)	17 (22)	0	15–25	NA	NA
% energy carbohydrate	40.0 (34.8, 44.0)	61 (80)	0	45–65	45–60	45–65
% energy total fat	37.8 (34.1, 42.7)	1 (1)	52 (68)	20–25	25–35	20–35
% energy saturated + trans-fat	15.5 (12.7, 19.1)		67 (88)	<10%	NA	10–35
Protein (g/day)	92.6 (81.7, 106.5)			EAR 54 g	0–6 months postpartum ^5^ AR (+) 15 g, PRI (+) 19 g >6 months postpartum ^5^ AR (+) 10 g, PRI (+) 13 g	NA
Fibre (Englest) (g)	29.0 (21.3, 33.6)	42 (55)		AI 30 g/day	AI 25 g	28 g

EAR = Estimated Average Requirement; AI = Adequate Intake; AR = Average Requirement; PRI = Population Reference Intake; NA = Not Available as the information is unavailable in the relevant recommendation documents. ^1^ Australia and New Zealand nutrient reference values. ^2^ European Food Safety Authority—dietary reference values. ^3^ National Institute of Health—nutrient recommendations and databases. ^4^ The value should be added to the AR of non-pregnant, non-lactating women. There is no PRI for energy, as it would be inappropriate to recommend energy intakes that exceed many individuals’ requirements. This would lead to a positive energy balance and promote weight gain. ^5^ The value should be added to the AR/PRI of non-pregnant, non-lactating women.

## Data Availability

The data presented in this study are available on request from the corresponding author. The data are not publicly available due to human ethics.

## Appendix A

**Table A1:** Median intakes of micronutrients and comparison to the nutrient reference values for Australia and New Zealand (breastfeeding women aged 19–50 years).

	MINI Study		Reference Recommendations					
			Australia and New Zealand ^1^		EFSA ^2^		NIH ^5^	
	Median Intake (25th, 75th)	n (%) <EAR/AI	EAR/AI	RDI	AR ^3^	PRI ^4^	EAR	RDA
Total Vitamin A (µg)	883 (642, 1214)	31 (41)	800	1100	1020 µg RE/day	1300 µg RE/day	900	1100
Thiamin (mg)	1.6 (1.2, 2.4)	20 (26)	1.2	1.4	0.072 mg/MJ	0.1 mg/MJ	1.2	1.4
Riboflavin (mg)	2.3 (1.9, 3.3)	2 (3)	1.3	1.6	1.7	2.0	1.3	1.6
Niacin Equivs. (mg)	38.0 (31.5, 46.0)	0	13	17	1.3 mg/MJ	1.6 mg/MJ	13	17
Vitamin B12 (µg)	4.6 (3.1, 6.5)	7 (9)	2.4	2.8	2.4		2.4	2.8
Folate DFE (µg)	519 (362, 760)	32 (42)	450	500	380	500	450	500
Vitamin C (mg)	100 (65, 155)	18 (24)	60	85	140	155	100	120
Vitamin D (µg)	4.9 (2.6, 8.2)	40 (53)	5		NA	NA	10	15
Vitamin E (mg)	10.2 (7.8, 13.5)	42 (55)	11	14	NA	NA	16	19
Calcium (mg)	949 (765, 1176)	25 (33)	840	1000	860	1000		1000
Copper (mg)	1.7 (1.4, 2.1)	25 (33)	1.5		NA	NA	1.0	1.3
Iron (mg)	14.4 (11.5, 20.3)	1 (1)	6.5	9	7	16	6.5	9
Magnesium (mg)	402 (327, 454)	8 (11)	255/265 ^6^	310/320 ^6^	NA	NA	255/265 ^6^	310/320 ^6^
Manganese (µg)	5477 (4119, 6827)	32 (61)	5000		NA	NA	NA	2600
Phosphorus (mg)	1616 (1342, 1826)	0	580	1000	NA	NA	580	700
Potassium (mg)	3478 (2900, 3952)	26 (34)	3200	4700 ^7^	NA	NA	NA	2800
Selenium (mg)	62.2 (50.8, 84.7)	42 (55)	65	75	NA	NA	59	70
Sodium (mg)	2978 (2192, 3972)	0	460–920	1600 ^7^	2000		NA	1500
Zinc (mg)	11.6 (9.6, 15.5)	21 (28)	10	12	8.6	10.4	10.4	13

EAR = Estimated average requirement; AI= adequate intake; RDI = recommended dietary intake; AR = average requirement; PRI = population reference intakes; RDA = recommended dietary allowance; DFE = dietary folate equivalents.; NA = Not Available as the information was unavailable from the relevant recommendations.^1^ Australia and New Zealand nutrient reference values [23]. ^2^ European Food Safety Authority–dietary reference values [49]. ^3^ The average requirement (AR) refers to the intake of a nutrient that meets the daily needs of half the people in a typical healthy population. ^4^ The population reference intake (PRI) is the intake of a nutrient likely to meet the needs of almost all healthy people [50]. ^5^ National Institute of Health–dietary reference intake [51]. ^6^ Magnesium EAR/RDI is 265/310 mg for 18–30 years and 265/320 mg for 31–50 years. ^7^ Suggested dietary target for potassium and sodium.
